# Belief Function Based Decision Fusion for Decentralized Target Classification in Wireless Sensor Networks

**DOI:** 10.3390/s150820524

**Published:** 2015-08-19

**Authors:** Wenyu Zhang, Zhenjiang Zhang

**Affiliations:** School of Electronic and Information Engineering, Key Laboratory of Communication and Information Systems, Beijing Municipal Commission of Education, Beijing Jiaotong University, Beijing 100044, China; E-Mail: zhangwenyu@bjtu.edu.cn

**Keywords:** decision fusion, distributed classification fusion, belief function, evidence theory, wireless sensor networks

## Abstract

Decision fusion in sensor networks enables sensors to improve classification accuracy while reducing the energy consumption and bandwidth demand for data transmission. In this paper, we focus on the decentralized multi-class classification fusion problem in wireless sensor networks (WSNs) and a new simple but effective decision fusion rule based on belief function theory is proposed. Unlike existing belief function based decision fusion schemes, the proposed approach is compatible with any type of classifier because the basic belief assignments (BBAs) of each sensor are constructed on the basis of the classifier’s training output confusion matrix and real-time observations. We also derive explicit global BBA in the fusion center under Dempster’s combinational rule, making the decision making operation in the fusion center greatly simplified. Also, sending the whole BBA structure to the fusion center is avoided. Experimental results demonstrate that the proposed fusion rule has better performance in fusion accuracy compared with the naïve Bayes rule and weighted majority voting rule.

## 1. Introduction

In wireless sensor detection and classification applications, decision fusion has attracted great interests for its advantages in combining the individual decisions into a unified one without sending raw data to the fusion center [1]. It provides a flexible solution for improving the classification accuracy without considering the classifiers used in local sensors [2]. Besides, the data transmission amount is greatly decreased, thus energy consumption and bandwidth demand are significantly reduced [3,4]. Yet decision fusion has been proven valuable in both civilian [5] and military [6] applications for its advantages in survivability, communication bandwidth, and reliability considerations.

Target classification is a common problem in applications of sensor networks. In decentralized target classification systems with decision fusion, each sensor independently conducts classification operation and uploads its local decision to the fusion center, which combines these decisions into a global one. Compared with target classification with a single sensor, multiple sensor decision fusion has better performance in classification accuracy, anti-noise, and reliability [7].

Fundamentally, multiclass decision fusion in WSNs is a problem of combining the ensemble decisions of several different classification systems. Existing methodologies can be concluded into two categories: hard decision (HD) fusion [8] and soft decision (SD) fusion [9]. In HD schemes, each sensor sends their hard decisions to fusion center, *i.e.*, clearly declare which class the target belongs to. The fusion center makes a decision according to some fusion rules, like counting rules [10], weighted sum [11], Neyman–Pearson criterion [12], or the max-log fusion [13]. The typical fusion HD scheme is the majority voting rule [14], though it has great advantage in easy implementation, the low fusion accuracy decreases it practicability. In SD schemes, local decisions are usually represented by values between 0 and 1 and the fusion operation is always conducted based on some decision fusion theories, including Bayesian fusion [15], Fuzzy logic [16] and belief function theory [17]. Except the above mentioned fusion schemes, many other centralized fusion approaches have been proposed, such as Decision Template [18], Bagging [19], and Boosting [20]. Some centralized fusion approaches, like Bagging and Boosting, have been proven to always perform better than other decentralized classifier ensemble approaches. However, centralized fusion approaches require sensor nodes to send raw data to the fusion center, a way consumes two much energy in data transmission, thus it is not applicable in decentralized target classification scenario in WSNs.

Another promising way to improve fusion performance is designing decision fusion schemes with Multiple-Input Multiple-Output (MIMO) technique, which enables sensors to transmit data to the fusion center via multiple access channels [21,22]. Benefit from the diversity gain in the fusion center, these MIMO based fusion schemes have been proven to have much better performance in sensing performance [23,24], anti-fading [25,26,27], bandwidth demand [28], and energy efficiency [29,30,31]. Even so, in MIMO based schemes, fundamental fusion rules underlying the decision fusion operation still play a central role in determining the overall sensing performance in the fusion center. Moreover, decision fusion in WSNs are usually designed based on wireless signal detection and transmission models [32,33,34,35], thus they may not be compatible with the multiclass classifier decision fusion problems.

As such, in this paper, we aim to design a decentralized decision fusion rule to improve overall classification performance while uploading data as little as possible. We focus on using belief function theory to address the decentralized decision fusion problem in WSNs with ideal error-free reporting channels. The belief function theory, also known as the Dempster-Shafer (DS) evidence theory, provides a flexible solution dealing with multisource information fusion problems, especially problems with uncertainty [36]. However, existing belief function based approaches have the two following disadvantages in practical applications:

(1) Poor compatibility with other classifiers. Different classification algorithms have their own advantages. It is hard to say which one is the best choice for a specific task, thus different classifiers may be used in different sensors, especially in heterogeneous WSNs. However, the prerequisite of applying belief function to addressing the information fusion problem is constructing rational basic belief assignments (BBAs), which are always constructed by specifically designed mass constructing algorithms, but have no business with other classification algorithms.

(2) Complex combination operation and energy inefficiency. The BBA combination operation is the key capacity enabling belief function theory dealing with fusion problems. However, the complex BBA combination operation requires each sensor node to upload the whole BBA structure to the fusion center, a way that consumes higher energy in data transmission than other fusion schemes, especially compared with HD fusion schemes. Moreover, the complex computation of combination operation adds the burden in system overhead to sensors and fusion center.

In conclusion, the main contributions include the following three aspects:

(1) A BBA construction algorithm based on the training output confusion matrix and decision reliability is proposed. The proposed mass construction algorithm has a strong compatibility without considering the classifiers used in the classification process. Compared with the probability-only based fusion schemes, the proposed approach is more reasonable because the constructed BBAs are adjusted by real-time observations.

(2) A new decision fusion rule based on belief function theory is proposed. By using Dempster’s combinational rule, we derive the explicit expression of the unified BBA in fusion center, and then a new simple fusion rule is derived. As a result, the complex BBA combination operation is avoided. Also, energy consumption for data transmission is reduced because there is no need to upload the whole BBA structure to fusion center.

(3) We test the proposed fusion rule with both a randomly generated dataset and a vehicle classification dataset. Experimental results show the proposed rule outperforms the weighted majority voting and naïve Bayes fusion rules.

The remainder of this paper is organized as follows: Section 2 gives a brief introduction of preliminaries of belief function theory. The proposed belief function based decision fusion approach is presented in Section 3. Section 4 provides the experimental results along with the analysis. Finally Section 5 concludes this paper.

## 2. Basics of Belief Function Theory

Belief function, also known the Dempster-Shafer evidence theory, provides a flexible framework for dealing with data fusion problems [37]. In general, the belief function based decision fusion framework mainly includes two phases: mass construction and BBA combination.

### 2.1. Mass Construction

In belief function, the frame of discernment is defined as a finite non-empty set and it is mutually exclusive and exhaustive. Let $\text{Ω}=\left\{A_{1},\cdots ,A_{c}\right\}$ be the frame of discernment and its corresponding power set is $2^{\text{Ω}}$. The mass function of $2^{\text{Ω}}$ is a function *m*: $2^{\text{Ω}}\to \left[0,1\right]$ and it satisfies the following condition

(1) $$\sum_{A\subset 2^{\text{Ω}}}m(A)=1\text{ and }m(Ø)=0$$

where *A* is a subset of $2^{\text{Ω}}$ and m(A) is called the basic belief assignment (BBA) representing the credible degree of subset *A*. There are two measures that characterize the credibility of hypothesis *A*, which are given by

(2) $$Bel(A)=\sum_{A_{i}\subset A}m(A_{i})$$

(3) $$Pls(A)=\sum_{A_{i}\cap A\neq Ø}m(A_{i})$$

Quantity Bel(A) can be interpreted as the support degree of hypothesis *A* of the evidence, while quantity Pls(A) can be interpreted as the degree not contradictory to *A* for the evidence. It is apparent that   Bel(A)≤Pls(A). In general, there are no unified frameworks or paradigms for mass construction. Any functions or algorithms transferring the observations into rational BBAs satisfying Equations 1–3 can be used as the BBA construction methods.

### 2.2. BBA Combination

One of the advantages of belief function being widely used in data fusion applications relies on its combinational rule enables to combine several independent BBAs into a unified one. Let ⊕ denotes the combination operator, for *M* independent BBAs, the combined BBA is $\;m={⊕}_{i=1}^{M}m_{i}$. According to Dempster’s combinational rule, the unified BBA of hypothesis *A* is calculated by [38]

(4) $$m(A)=\frac{\sum_{\cap_{j=1}^{M}A_{j}=A}\prod_{i=1}^{M}m(A_{i})}{1-\kappa }$$

where

(5) $$\kappa =\sum_{\cap_{j=1}^{M}A_{j}=Ø}\prod_{i=1}^{M}m(A_{i})$$

and it is called the conflict of the *M* BBAs. It also can be regarded as a normalization factor in Equation (4). If the conflict κ is approximated to 1, it indicates that a high conflict degree exists among the combining BBAs, and the fusion results may be unreliable in practice. Therefore, the mass construction method must avoid the situations that high conflicts exist among the obtained BBAs. With the obtained unified BBA, the final decision can be made by choosing the class label with maximum pignistic probability, which is calculated by [39]

(6) $$BetP(A)=\sum_{A_{i}\subset A}\frac{m(A)}{\left|A_{i}\right|}$$

## 3. Belief Function Based Multi-Class Decision Fusion

### 3.1. System Model

**Figure 1:**
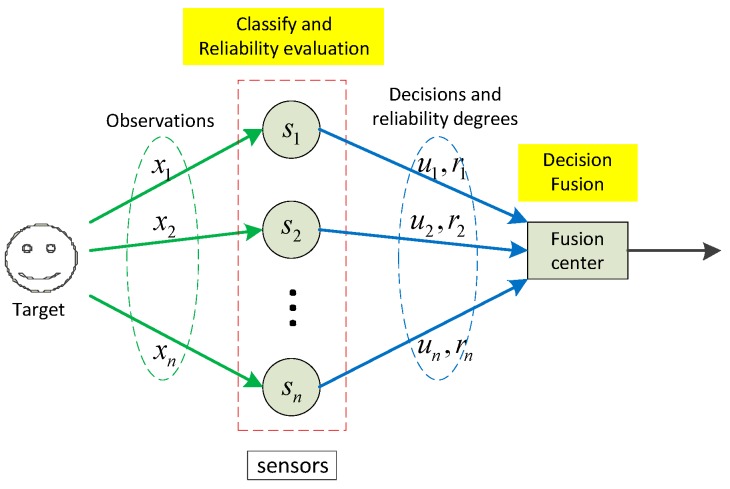
System model of the proposed decision fusion approach.

The system model is depicted in Figure 1. Suppose there is a distributed sensor network with $s=\left\{s_{1},\cdots ,s_{n}\right\}$ sensors. All sensors are assumed to be mutually independent and they can use any classifiers for the classification task. For a target with $\text{Θ}=\left\{\omega_{1},\cdots ,\omega_{c}\right\}$ possible classes (labels), the *n* sensors conduct local classification operations according to their own observations $x=\left\{x_{1},\cdots ,x_{n}\right\}$, and we set the corresponding hard decisions are $u=\left\{u_{1},\cdots ,u_{n}\right\}$, in which $u_{i}\subset \text{Θ }\left(1\leq i\leq n\right)$. Also, we define the reliability degrees of the decisions as $r=\left\{r_{1},\cdots ,r_{n}\right\}$, which can be computed according to the corresponding real-time observations $x=\left\{x_{1},\cdots ,x_{n}\right\}$. With the received hard decisions and reliability degrees, the fusion center then conducts the decision fusion operation with the proposed fusion rule. At last, the final decision is made by choosing the class (label) with the maximum BBA. Note that the decision fusion operation in the fusion center is conducted according to a simple fusion rule induced by the belief function theory, thus the complex BBA construction and BBA combination operations are avoided. In the following subsections, the detailed local classification, reliability evaluation, and decision fusion processes will be provided.

### 3.2. Classification and Reliability Evaluation

In local sensors, the classification process can be made by any appropriate recognition algorithms. For a multi-class pattern recognition problem, we assume that all the local classifiers are well trained and the training output confusion matrices are previously known to the fusion center, *i.e.*, the fusion center maintains a confusion matrix for each sensor. We don’t consider the details of its classification operation, such as signal segmentation, feature extraction, and classification algorithm. For sensor $s_{i}\left(1\leq i\leq n\right)$, when given a new observation, it conducts the classification operation and makes it local decision $u_{i}$. For decision $u_{i}$, we define $r_{i}$ as its corresponding reliability degree. In this paper, we propose a distance based algorithm to calculate the reliability degree for each local decision.

The best way to calculate the reliability of a classifier’s output is designing a specific algorithm measuring the similarity of the output before the final decision is made [40]. For example, if we want to know the reliability of a local decision when the classifier is an artificial neural network (ANN), the output before decision making in the output layer can be used as the basis for reliability evaluation. For another example, when using *k*-NN classifier for classification, the distance between the object and *k* nearest neighbors in sample set of each class label can be exploited to measure the reliability.

Herein in this paper we also provide a more general method to evaluate the reliability degree for each local decision. The method follows the basic assumption that, when the object to be classified has a smaller distance to the sample set of a class label, then the decision result is more reliable. On the contrary, when the distance is large, the reliability is low. This distance can be computed by any appropriate distance definitions, such as Euclidean distance, Mahalanobis distance, Hamming distance, and the like. Also, the chosen samples for distance calculation can be the whole sample set, or the *k* nearest neighbors to the object. Usually, the distance definition is Euclidean distance and the chosen samples are one to five nearest neighbors to the object.

For a sensor $s_{i}$, denote its training set as $T_{i}=\left\{\left(y_{1}^{\left(i\right)},\omega_{1}\right),\cdots ,\left(y_{c}^{\left(i\right)},\omega_{c}\right)\right\}$, where $y_{k}^{\left(i\right)}\left(1\leq k\leq c\right)$ is a *N*-dimensional vector containing *N* data samples. Given a new observation $x_{i}$, the distance to each sample set can be calculated and we denote $d_{i,j}$ as the distance between $x_{i}$ and sample set $y_{j}$. Let the local decision $u_{i}=\omega_{k}\left(1\leq k\leq c\right)$ and its corresponding distance is $d_{i,k}$, we define the relative distance $\nabla d_{i,j}$ as

(7) $$\nabla d_{i,j}=\frac{d_{i,j}}{d_{i,k}},1\leq j\leq c,j\neq k$$

**Figure 2:**
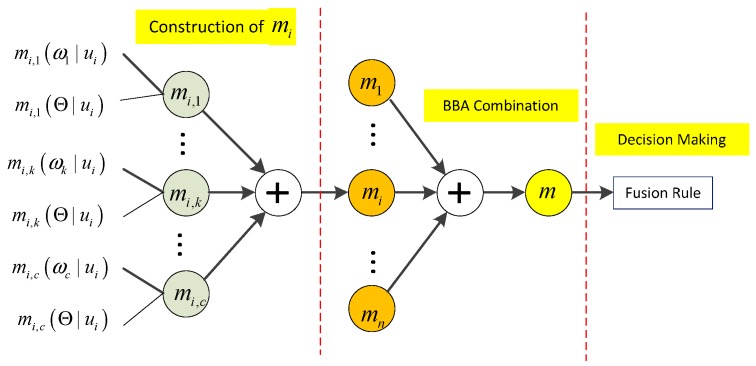
Derivation process of the proposed approach, in which ⊕ denotes the Dempster BBA combination operation, $m_{i}\left(1\leq i\leq n\right)$ is the constructed BBA of sensor $s_{i}$, m is the global BBA of all sensors.

If the relative distance $d_{i,j}$ is large, it means that we have sufficient confidence to confirm that $\omega_{j}$ is not the class label of the target. On the contrary, if $d_{i,j}$ is small, the possibility that $\omega_{j}$ is class label will be large. By using an exponential function, the distance can be transferred into BBAs [41]. Also, we use an exponential function to map distance into reliability. Similar to the transferring function in [41], we define the reliability measurement of decision $u_{i}$ as

(8) $$r_{i}=\underset{1\leq j\leq c,j\neq k}{\min}\left\{\lambda \left(1-\exp\left(-\beta \nabla {d_{i,j}}^{2}\right)\right)\right\}$$

where β and λ are positive constants and they are associated to the relative distance. Together with the local decision $u_{i}$, obtained reliability measurement $r_{i}$ will be uploaded to the fusion center. In the fusion center, the received pattern $\left(u_{i},r_{i}\right)$ will be used as the basis for the global decision making. In next subsection, we will elaborate the detailed derivation of the proposed decision fusion rule, including BBA construction, BBA combination and decision making, as illustrated in Figure 2.

### 3.3. BBA Construction

Reasonable BBAs are the prerequisite when applying belief function to address data fusion problems. With the received patterns $O=\left\{\left(u_{1},r_{1}\right),\cdots ,\left(u_{n},r_{n}\right)\right\}$ from sensors, a set of probability vectors can be obtained from the corresponding confusion matrix of sensor $s_{i}$. For decision $u_{i}$, we have the probability vector $P_{i}=\{p_{i}(u_{i}|\omega_{1}),\;\;\cdots ,\;\;p_{i}(u_{i}|\omega_{c})\}$, in which $p_{i}\left(u_{i}\text{|}\omega_{k}\right)\;\left(1\leq i,k\leq c\right)$ is the conditional probability of class label $\omega_{k}$ when the local decision is $u_{i}$. Although belief and probability are two different concepts, but one thing is certain that, a larger probability will be accompanied by a larger belief. In the contrary, a smaller belief value corresponds to smaller probability value. This distinct evidence can be postulated to transfer each probability $p_{i}\left(u_{i}\text{|}\omega_{k}\right)$ into a BBA $m_{i,k}\left(u_{i}\text{|}\omega_{k}\right)$ over the frame of discernment $\text{Θ}=\left\{\omega_{1},\cdots ,\omega_{c}\right\}$, as given by

(9) $$m_{i,k}\left(u_{i}|\omega_{k}\right)=r_{i}p_{i}\left(u_{i}|\omega_{k}\right)$$

for the compound class Θ, we define its BBA as

(10) $$m_{i,k}\left(u_{i}|\Theta \right)=1-r_{i}p_{i}\left(u_{i}|\omega_{k}\right)$$

thus for any other classes $\text{A}\subset 2^{\text{Θ}}\backslash \left\{\omega_{k},\text{Θ}\right\}$, their BBAs equal to 0, that is

(11) $$m_{i,k}\left(u_{i}|A\right)=0,\forall A\subset 2^{\Theta }\backslash \{\omega_{k},\Theta \}$$

With the obtained BBAs $\left\{m_{i,1},\cdots m_{i,c}\right\}$, the BBA $m_{i}$ with respect to $P_{i}$ can be calculated by

(12) $$m_{i}\left(u_{i}|\omega_{k}\right)={⊕}_{k=1}^{c}m_{i,k}\left(u_{i}|\omega_{k}\right)$$

where ⊕ denotes the BBA combination operation. For convenience, we denote $p_{i}\left(u_{i}\text{|}\omega_{k}\right)$ as $p_{i,k}$ for short. Note that the value of $\overset{c}{\underset{k=1}{\sum }}p_{i,k}$ always not equals to 1, *i.e.*, for a decision, the sum of probability of detection and probability of false alarm does not equal 1. According to Dempster’s combinational rule, the explicit expression of $m_{i}$ is given by

(13) $$m_{i}\left(u_{i}|\omega_{k}\right)=\frac{1}{1-\kappa_{i}}\frac{r_{i}p_{i,k}}{1-r_{i}p_{i,k}}\prod_{j=1}^{c}\left(1-r_{i}p_{i,j}\right)$$

(14) $$m_{i}\left(u_{i}|\Theta \right)=\frac{1}{1-\kappa_{i}}\prod_{j=1}^{c}\left(1-r_{i}p_{i,j}\right)$$

where $\kappa_{i}$ designates the conflict degree of BBAs $\left\{m_{i,1},\cdots m_{i,c}\right\}$, and it equals to

(15) $$\kappa_{i}=1-\left(\sum_{k=1}^{c}\frac{r_{i}p_{i,k}}{1-r_{i}p_{i,k}}+1\right)\prod_{j=1}^{c}\left(1-r_{i}p_{i,j}\right)$$

Combined with Equations (11) and (12), we have the following relationship between BBA $m_{i}\left(\omega_{k}\right)$ and m(Θ)

(16) $$m_{i}\left(\omega_{k}\right)=\frac{r_{i}p_{i,k}}{1-r_{i}p_{i,k}}m_{i}\left(\Theta \right)$$

### 3.4. BBA Combination

After the BBA construction process, we obtained BBAs $𝓜=\left\{m_{1},\cdots ,m_{n}\right\}$. The next step is combining these BBAs into a unified one. We assume that all BBAs in 𝓜 are mutually independent, given two BBAs $m_{i}\left(1\leq i\leq c\right)$ and $m_{j}\left(1\leq j\leq c\right)$, for class label $\omega_{k}\subset \text{Θ}$, we have

(17) $$\begin{array}{l} m_{i}⊕m_{j}\left(\omega_{k}\right)=\frac{1}{1-\kappa_{i,j}}(\frac{r_{i}p_{i,k}}{1-r_{i}p_{i,k}}m_{i}\left(\Theta \right)\frac{r_{i}p_{j,k}}{1-r_{j}p_{j,k}}m_{j}\left(\Theta \right)\\ \;+\frac{r_{i}p_{i,k}}{1-r_{i}p_{i,k}}m_{i}\left(\Theta \right)m_{j}\left(\Theta \right)+\frac{r_{j}p_{j,k}}{1-r_{j}p_{i,k}}m_{j}\left(\Theta \right)m_{i}\left(\Theta \right))\\ \;=\frac{1}{1-\kappa_{i,j}}\left(\frac{1}{\left(1-r_{i}p_{i,k}\right)\left(1-r_{j}p_{i,k}\right)}-1\right)m_{i}\left(\Theta \right)m_{j}\left(\Theta \right) \end{array}$$

For compound class Θ, we have

(18) $$m_{i}⊕m_{j}\left(\Theta \right)=\frac{1}{1-\kappa_{i,j}}m_{i}\left(\Theta \right)m_{j}\left(\Theta \right)$$

Equations (17) and (18) indicate that, when given *n* BBAs, the combined result follows a certain rule. Thus we have reasons to assume that the unified combination result in the fusion center is

(19) $$m(\omega_{k})=\frac{1}{1-\kappa }\left(\prod_{i=1}^{n}\frac{1}{\left(1-r_{i}p_{i,k}\right)}-1\right)\prod_{i=1}^{n}m_{i}(\Theta )$$

(20) $$m(\Theta )=\frac{1}{1-\kappa }\prod_{i=1}^{n}m_{i}(\Theta )$$

*Proof*: The above proposition can be proved via mathematical proof of induction. Apparently, given *n*+1 sensors, we have

(21) $$m(\Theta )=\frac{1}{1-\kappa }\prod_{i=1}^{n+1}m_{i}(\Theta )$$

Then we just have to prove Equation (19) is true for any sensor number. Assume that Equation (17) is true with *n* sensors, when sensor number is *n* + 1, we have

(22) $$\begin{array}{l} m(\omega_{k})=\frac{1}{1-\kappa }(\left(\prod_{i=1}^{n}\frac{1}{\left(1-r_{i}p_{i,k}\right)}-1\right)\prod_{i=1}^{n}m_{i}(\Theta )\frac{r_{n+1}p_{n+1,k}}{1-r_{n+1}p_{n+1,k}}m_{n+1}\left(\Theta \right)\\ \;+\left(\prod_{i=1}^{n}\frac{1}{\left(1-r_{i}p_{i,k}\right)}-1\right)\prod_{i=1}^{n}m_{i}(\Theta )m_{n+1}\left(\Theta \right)+\frac{r_{n+1}p_{n+1,k}}{1-r_{n+1}p_{n+1,k}}m_{n+1}\left(\Theta \right)\prod_{i=1}^{n}m_{i}(\Theta ))\\ \;=\frac{1}{1-\kappa }\left(\prod_{i=1}^{n+1}\frac{1}{\left(1-r_{i}p_{i,k}\right)}-1\right)\prod_{i=1}^{n+1}m_{i}(\Theta ) \end{array}$$

Consequently, we have proved that equation is true with any sensor number.

### 3.5. Decision Making

In the above subsection, we have derived the explicit expression of the unified BBA in the fusion center, as given in Equations (19) and (20). The final decision can be made by choosing the label with maximum belief assignment, as given by

(23) $$\omega_{d}=\arg\underset{1\leq k\leq c}{\max}\left\{\frac{1}{1-\kappa }\left(\prod_{i=1}^{n}\frac{1}{\left(1-r_{i}p_{i,k}\right)}-1\right)\prod_{i=1}^{n}m_{i}(\Theta )\right\}$$

Actually, there is no need to consider the conflict degree κ because it is the same for all class labels, thus the above decision rule can be simply expressed as

(24) $$\omega_{d}=\arg\underset{1\leq k\leq c}{\min}\left\{\prod_{i=1}^{n}\left(1-r_{i}p_{i,k}\right)\right\}=\arg\underset{1\leq k\leq c}{\min}\left\{\prod_{i=1}^{n}m_{i,k}\left(\Theta |u_{i}\right)\right\}$$

Also, the above decision making rule is equivalent to

(25) $$\omega_{d}=\arg\underset{1\leq k\leq c}{\min}\left\{\sum_{i=1}^{n}\log\left(1-r_{i}p_{i,k}\right)\right\}$$

With the above decision making rule, the complex BBA combination operation is avoided, thus the system overhead is reduced. The pseudocode of the proposed approach is shown in the Algorithm 1. Note that the classification performance, *i.e.*, the training confusion matrix of each local sensor is default known to the fusion center. This may be realized by sending the confusion matrix to fusion after the training process. Another way is that the classifiers and sample data can be previously trained in the fusion center before they are embedded into the sensors, thus the classification performances of the sensors are also known to the fusion center.

**Algorithms 1** Belief function based decentralized classification fusion for WSN1:  **event** target is detected by *n* sensors **do**
2:    **for each** observation $x_{i}\left(1\leq i\leq n\right)$ is received by sensor $s_{i}$
**do**
3:        classify the object and obtain local decision $u_{i}$
4:        calculate local reliability measurement $r_{i}$ by (8)
5:        send pattern $\left(u_{i},r_{i}\right)$ to fusion center
6:    **end for each**
7:  **end event**
8:  
9:  **event** fusion center receives uploading from sensors **do**
10:   **for each** received pattern $\left(u_{i},r_{i}\right)$
**do**
11:        find the probability vector $P_{i}=\left\{p_{i,1},\cdots ,p_{i,c}\right\}$
12:   **end for each**
13:   make final decision $\omega_{d}\leftarrow \text{arg}\underset{1\leq k\leq c}{\min}\left\{\overset{n}{\underset{i=1}{\prod }}\left(1-r_{i}p_{i,k}\right)\right\}$
14:  **end event**

## 4. Experimental Results

In experimental section, two experiments will be conducted. The first one is used to evaluate the fusion performance by using a randomly generated dataset, whose sensor number and the sensors’ classification accuracies can be artificially changed. Therefore, the performance comparison results can be provided with changing sensor number or sensor accuracy. The next one is testing the performance of the proposed fusion approach by using the sensit vehicle classification dataset [42]. In the two experiments, all sensor nodes are all assumed to be equipped with sufficient computational capacity to underlay the local classification and reliability evaluation operation. We assume that the reporting channel is ideally an error-free channel. Also, we don’t consider how to quantify the reliability degree when it is transmitted to the fusion center. Thus the information of each sensor will be sent to the fusion center without distortion.

Considering the computation complexity, the following two easy implementing algorithms are used as the local classifiers: *k*-nearest neighbors (*k*-NN) algorithm and extreme learning machine (ELM) neural network. The detailed introduction of k-NN and ELM algorithms can be found in [43,44], respectively. For performance comparison, the following two conventional decision fusion approaches will be used.

*Naïve Bayes*: the naïve Bayes fusion method assumes that all decisions are mutually independent. In binary fusion systems, this fusion method is regarded as the optimal fusion rule. In a fusion system with *M* sensors, denote $p_{i,k}$ as the probability of label *k* corresponding to decision $u_{i}$, the fusion decision is made by choosing the label with maximum fusion statistic, as given by

(26) $$l_{d}=\arg\underset{1\leq k\leq c}{\max}\left\{\prod_{i=1}^{n}p_{i,k}\right\}$$

*Weighted majority voting*: denote $u_{i,k}\left(1\leq i\leq n,1\leq k\leq c\right)$ as the decision on label $\omega_{k}$ of sensor $s_{i}$. When the target belongs to $\omega_{k}$, we have $u_{i,k}=1$ and $u_{i,j}=0\;\;\left(1\leq j\leq c,j\neq k\right)$. In weighted majority voting, decision $u_{i,k}$ is weighted by an adjusting coefficient $b_{i}$ , and the decision is made by

(27) $$l_{d}=\arg\underset{1\leq k\leq c}{\max}\left\{\sum_{i=1}^{n}b_{i}u_{i,k}\right\}$$

weight $b_{i}$ can be calculated by

(28) $$b_{i}\propto \log\left(\frac{p_{i}}{1-p_{i}}\right)$$

where $p_{i}$ is the classification accuracy of sensor $s_{i}$. Apparently, a sensor with higher accuracy will be assigned a larger weight. Always, this rule performs better than the simple majority voting rule.

### 4.1. Experiment on Randomly Generated Dataset

In this test, our goal is to evaluate the performance variation of the three fusion approaches with different sensor numbers or local classification accuracies. Since the local classification accuracies of datasets in reality are fixed, the randomly generated the dataset must be used if we want to evaluate the performance with changing sensor classification accuracies. In this test, we randomly generated the dataset by using Gaussian random number generation function. The target class label number is fixed as five, each sample data is assumed to have two randomly generated attributes following different Gaussian distributions.

**Figure 3:**
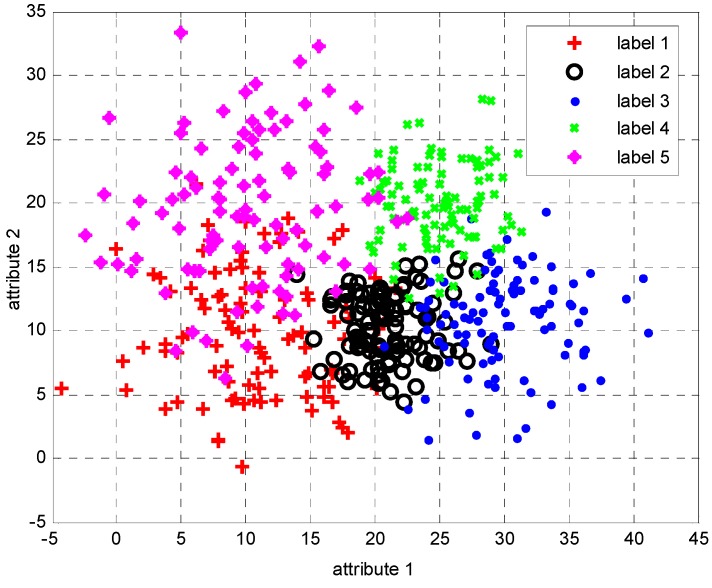
Example of a randomly generated dataset, each class label has 100 samples and the coefficient α equals to 1.

As shown in Table 1, α is a coefficient changing the standard deviations of the sensor data attributes. For example, the two attributes of class label $\omega_{3}$ follow the two Gaussian probability density functions (pdf): 𝒩(30,4α) and 𝒩(10,4α), respectively. Apparently, coefficient α determines the sensor classification accuracies, *i.e.*, a larger α brings lower classification accuracy. Figure 3 gives an example depiction of the randomly generated sample data.

**Table 1:** Data generation parameters.

Label	$\mu_{1}$	$\mu_{2}$	σ
$\omega_{1}$	10	10	5α
$\omega_{2}$	20	10	3α
$\omega_{3}$	30	10	4α
$\omega_{4}$	25	20	3α
$\omega_{5}$	10	20	5α

Since the dataset is randomly generated each time, we repeat it i20 times to obtain the average classification accuracy. In each repetition, to know the posterior probabilities of the training process, 1500 samples and 500 samples are respectively generated as the training data set and validation data set, in which each class label has the same sample number, i.e. each of them has 300 train samples and 100 valid samples. After training process, the classifier used in each sensor is also obtained. Subsequently, 1000 samples are randomly generated as new observations. In these new observations, the class label of each observation is randomly selected, thus the number of each class label is approximated to 200. Next we classify the new observations by using the classifiers obtained in the training process. At the same time, the reliability degree of each decision is calculated by using Expression (8). Next, the local decisions and their corresponding reliability degrees are uploaded to fusion center and the final decision is finally made according to Equation (23).

As aforementioned, the following two classifiers are used for classification in sensors: *k*-NN and ELM neural network. If there are no specific instructions, the *k* nearest neighbors used in *k*-NN is 3. In the reliability evaluation process, the nearest neighbor number used for calculating distances is also fixed as 3. The number of hidden neurons in ELM is 50 and the activation function is “radbas” function. For the weighted majority voting rule, the weight of each decision is calculated by $b_{i}=\text{log}\left(\frac{2p_{i}}{1-p_{i}}\right)$. In Expression (8), parameter β is fixed as 1.5, and parameter $\lambda_{i}$ corresponding *i*th decision $u_{i}$ is calculated by

(29) $$\lambda_{i}=\frac{0.9}{\underset{1\leq j\leq 5}{\max}\left(p_{i,j}\right)}$$

**Figure 4:**
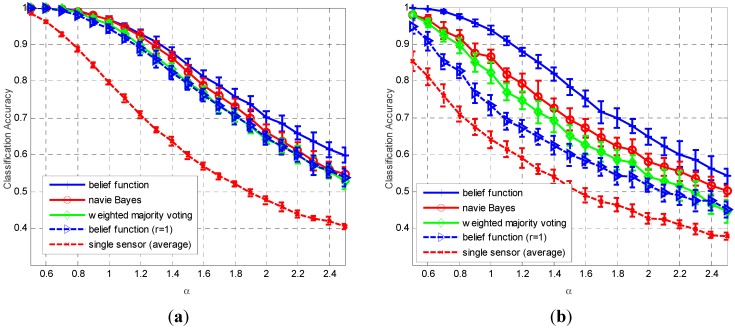
Average classification accuracy (plus and minus one standard deviation) as a function of α values, obtained by 20 repetitions. The sensor number is fixed as *M* = 5 and the used classifiers in subplots (**a**,**b**) are *k*-NN and ELM neural network, respectively.

The following three approaches are used for performance comparison: the proposed belief function fusion approach, naïve Bayes fusion, and majority voting fusion. Define classification accuracy as the total number of correct classifications over the number of trials. The classification accuracy results with changing α values are shown in Figure 4. The used classifiers in Figure 4a and Figure 4b are *k*-NN and ELM neural network, respectively. The sensor number is fixed as 5. In Figure 4a, when the value of coefficient α increases from 0.6 to 2.5, the average classification accuracies of the local sensors decrease from 0.97 to 0.4, along with the decreasing of the classification accuracies of fusion results. In Figure 4b, the average sensor classification accuracies and final fusion accuracies also decrease with the increasing of α value. We can find that the classification of the ELM neural network is usually lower than the *k*-NN classifier, especially when α is smaller than 1.4, thus obviously the classification accuracies of the three approaches when using ELM classifier are lower than the fusion accuracies of *k*-NN classifier. Apparently, we can observe that the proposed belief function based fusion approach always outperforms the naïve Bayes fusion and weighted majority voting fusion approaches, especially for the classifiers with lower classification performances.

**Figure 5:**
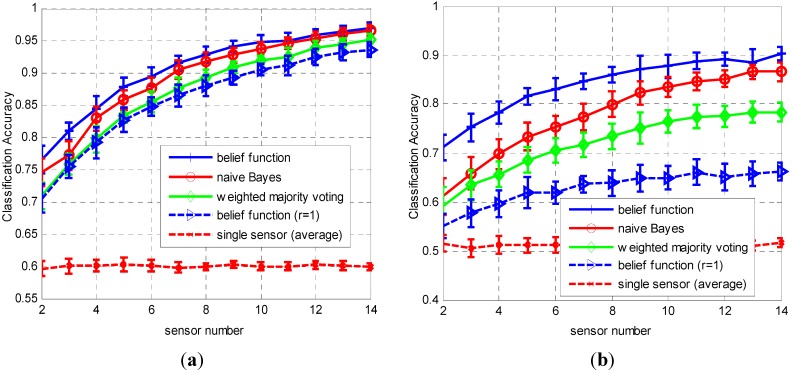
Average classification accuracy (plus and minus one standard deviation) as a function of sensor number, obtained by 20 repetitions. The value of coefficient α is fixed as 1.5 and the used classifiers in subplots (**a**,**b**) are *k*-NN and ELM neural network, respectively.

The performance comparison results with changing sensor numbers are plotted in Figure 5. In this test, the value of coefficient α is fixed as 1.5. The results also show that the proposed approach always outperforms than the other two approaches with changing sensor numbers. The accuracy improvement is more significant when sensor number is less than 7.

The proposed fusion approach has a very similar form to the naïve Bayes fusion rule, but they have distinct difference in fusion accuracies. As shown in Figure 4 and Figure 5, when the decision reliability in each sensor is fixed as 1, the classification accuracies of the fusion results are always lower than the other two approaches. This result indicates that the reliability evaluation method is the key factor influencing the fusion results’ classification accuracies of the proposed rule.

**Figure 6:**
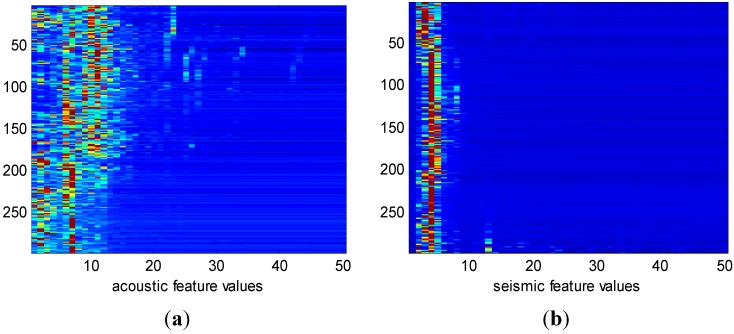
Image plot of extract features. Subplots (**a**,**b**) are features extracted from acoustic signals and seismic signals, respectively. The vehicle type is AAV and each of the subplots has 297 features.

### 4.2. Experiment on Vehicle Classification

In this test, we use the *sensit vehicle classification dataset* collected in real application, in which the wireless distributed sensor networks are used for vehicle surveillance. There are 23 sensors deployed in total along the road side listening for passing vehicle. When vehicles are detected, the captured signal of the target vehicle is recorded for acoustic, seismic, and infrared modalities. The signal segmentation and feature extraction process can be found in [42]. In our test, 11 sensor nodes are selected for vehicle classification. The target vehicle may belong to the following two types: Assault Amphibian Vehicle (AAV) and DragonWagon (DW). Features extracted from the recorded acoustic and seismic signals are used for vehicle classification. Examples of the extracted features are shown in Figure 6.

**Figure 7:**
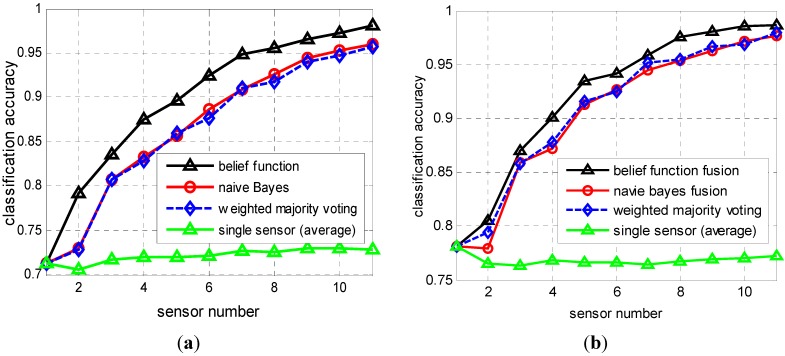
Classification accuracy as a function of sensor numbers. Classifiers used in subplots (**a**,**b**) are *k*-NN and ELM neural network, respectively.

The experiment procedure is the same with the previous experiment, thus we don’t repeat it again. The difference is that, when the training samples are given, the classification accuracy of sensor nodes is fixed as a constant value. In this test, the “*k*” used in *k*-NN classifier and reliability calculation are all equal to 1. The two parameters λ and β in Expression (8) are fixed as 1 and −0.5, respectively. The hidden neuron number of ELM neural network is 50 and the activation function is also the “radbas” function. The accuracy comparison of fusion results are provided in Figure 7. We can observe that the performance improving of the proposed approach for *k*-NN classifier is better than the ELM classifier. But the final fusion accuracy of ELM is higher than *k*-NN classifier when the sensor number is the same. Again, we easily conclude that the proposed approach has better performance in improving the fusion accuracy for distributed target classification applications.

## 5. Conclusions

In this paper we focus on the decentralized classification fusion problem in WSNs and a new simple but effective decision fusion rule based on belief function theory is proposed. We propose a distance based approach to evaluate the decision reliability of each sensor. Then the detailed derivation process of the proposed approach is illustrated, including BBA construction, BBA combination, and decision making. The experimental results demonstrate that the proposed fusion rule has better performance in fusion accuracy compared with the naïve Bayes fusion and weighted majority voting rules. Future study may include the following aspects: (1) finding better ways to calculate the decision reliability to improve the fusion accuracy; (2) designing specific solutions for classifier combination application, such as neural networks; (3) applying the proposed rule in other multi-class fusion applications, like remote sensing, image fusion, and multi-symbol signal modulation.

## References

[1] Khaleghi B., Khamis A., Karray F.O., Razavi S.N. (2013). Multisensor data fusion: A review of the state-of-the-art. Inf. Fusion.

[2] Lei A., Schober R. (2010). Multiple-symbol differential decision fusion for mobile wireless sensor networks. IEEE Trans. Wirel. Commun..

[3] Nakamura E.F., Loureiro A.A.F., Frery A.C. (2007). Information fusion for wireless sensor networks: Methods, models, and classifications. ACM Comput. Surv. (CSUR).

[4] Liu C.X., Liu Y., Zhang Z.J., Cheng Z.Y. (2013). High energy-efficient and privacy-preserving secure data aggregation for wireless sensor networks. Int. J. Commun. Syst..

[5] Salvo Rossi P., Ciuonzo D., Ekman T., Dong H. (2015). Energy detection for MIMO decision fusion in underwater sensor networks. IEEE Sens. J..

[6] Cho T., Lee C., Choi S. (2013). Multi-sensor fusion with interacting multiple model filter for improved aircraft position accuracy. Sensors.

[7] Zhang Z.J., Lai C.F., Chao H.C. (2014). A green data transmission mechanism for wireless multimedia sensor networks using information fusion. IEEE Wirel. Commun..

[8] Ahsant B., Viswanathan R., Jeyaratnam S., Jayaweera S.K. New results on large sample performance of counting rules. Proceedings of the IEEE 2012 50th Annual Allerton Conference on Communication, Control, and Computing (Allerton).

[9] Chen B., Jiang R., Kasetkasem T., Varshney P.K. (2004). Channel aware decision fusion in wireless sensor networks. IEEE Trans. Signal Process..

[10] Viswanathan R., Aalo V. (1989). On counting rules in distributed detection. IEEE Trans. Acoust. Speech Signal Process..

[11] Laitrakun S., Coyle E.J. Optimizing the collection of local decisions for time-constrained distributed detection in WSNs. Proceedings of the 2013 IEEE INFOCOM.

[12] Teneketzis D., Varaiya P. (1984). The decentralized quickest detection problem. IEEE Trans. Autom. Control.

[13] Lei A., Schober R. (2010). Coherent max-log decision fusion in wireless sensor networks. IEEE Trans. Commun..

[14] Faria F., dos Santos J.A., Torres R.D.S., Rocha A., Falcao A.X. Automatic fusion of region-based classifiers for coffee crop recognition. Proceedings of the 2012 IEEE International Geoscience and Remote Sensing Symposium (IGARSS).

[15] Makarenko A., Whyte H.D. (2006). Decentralized bayesian algorithms for active sensor networks. Inf. Fusion.

[16] Melin P., Castillo O. (2014). A review on type-2 fuzzy logic applications in clustering, classification and pattern recognition. Appl. Soft Comput..

[17] Shen B., Liu Y., Fu J.S. (2014). An integrated model for robust multisensor data fusion. Sensors.

[18] Kuncheva L.I., Bezdek J.C., Duin R. (2001). Decision templates for multiple classifier fusion: An experimental comparison. Pattern Recognit..

[19] Breiman L. (1996). Bagging predictors. Mach. Learn..

[20] Freund Y., Schapire R.E. (1997). Decision-theoretic generalization of on-line learning and an application to boosting. J. Comput. Syst. Sci..

[21] Jiang F., Chen J., Swindlehurst A.L., Lopez-Salcedo J.A. (2015). Massive MIMO for wireless sensing with a coherent multiple access channel. IEEE Trans. Signal Process..

[22] Nevat I., Peters G.W., Collings I.B. (2014). Distributed detection in sensor networks over fading channels with multiple antennas at the fusion centre. IEEE Trans. Signal Process..

[23] Ciuonzo D., Romano G., Salvo Rossi P. (2013). Performance analysis and design of maximum ratio combining in channel-aware MIMO decision fusion. IEEE Trans. Wirel. Commun..

[24] Salvo Rossi P., Ciuonzo D., Romano G. (2013). Orthogonality and cooperation in collaborative spectrum sensing through MIMO decision fusion. IEEE Trans. Wirel. Commun..

[25] Ciuonzo D., Salvo Rossi P., Dey S. (2015). Massive MIMO channel-aware decision fusion. IEEE Trans. Signal Process..

[26] Ciuonzo D., Romano G., Salvo Rossi P. Decision fusion in MIMO wireless sensor networks with channel state information. Proceedings of the IEEE 7th Sensor Array and Multichannel Signal Processing Workshop (SAM).

[27] Li F., Evans J.S., Dey S. (2011). Decision fusion over noncoherent fading multiaccess channels. IEEE Trans. Signal Process..

[28] Ciuonzo D., Romano G., Salvo Rossi P. (2012). Channel-aware decision fusion in distributed MIMO wireless sensor networks: Decode-and-fuse *vs.* decode-then-fuse. IEEE Trans. Wirel. Commun..

[29] Zhang X., Poor H.V., Chiang M. (2008). Optimal power allocation for distributed detection over MIMO channels in wireless sensor networks. IEEE Trans. Signal Process..

[30] Ciuonzo D., Romano G., Salvo Rossi P. (2013). Optimality of received energy in decision fusion over a Rayleigh fading diversity MAC with non-identical sensors. IEEE Trans. Signal Process..

[31] Salvo Rossi P., Ciuonzo D., Kansanen K., Ekman T. (2015). On energy detection for MIMO decision fusion in wireless sensor networks over NLOS fading. IEEE Commun. Lett..

[32] Berger C.R., Guerriero M., Zhou S., Willett P.K. (2009). PAC *vs.* MAC for decentralized detection using noncoherent modulation. IEEE Trans. Signal Process..

[33] Ciuonzo D., Papa G., Romano G., Salvo Rossi P., Willett P.K. (2013). One-bit decentralized detection with a Rao Test for multisensor fusion. IEEE Signal Process. Lett..

[34] Ciuonzo D., de Maio A., Salvo Rossi P. (2015). A systematic framework for composite hypothesis testing of independent Bernoulli Trials. IEEE Signal Process. Lett..

[35] Salvo Rossi P., Ciuonzo D., Ekman T. (2015). HMM-based decision fusion in wireless sensor networks with noncoherent multiple access. IEEE Commun. Lett..

[36] Ristic B., Smets P. (2005). Target identification using belief functions and implication rules. IEEE Trans. Aerosp. Electron. Syst..

[37] Aggarwal C.C., Yu P.S. (2009). A survey of uncertain data algorithms and applications. IEEE Trans. Knowl. Data Eng..

[38] Shafer G. (1976). A Mathematical Theory of Evidence.

[39] Smets P., Kennes R. (1994). The transferable belief model. Artif. Intell..

[40] Polikar R. (2006). Ensemble based systems in decision making. IEEE Circuits Syst. Mag..

[41] Zouhal L.M., Denœux T. (1998). An evidence-theoretic k-NN rule with parameter optimization. IEEE Trans. Syst. Man Cybern. Part C: Appl. Rev..

[42] Duarte M.F., Hu Y.H. (2004). Vehicle classification in distributed sensor networks. J. Parallel Distrib. Comput..

[43] Weinberger K.Q., Saul L.K. (2009). Distance metric learning for large margin nearest neighbor classification. J. Mach. Learn. Res..

[44] Huang G.B., Zhou H., Ding X., Zhang R. (2012). Extreme learning machine for regression and multiclass classification. IEEE Trans. Syst. Man Cybern. Part B: Cybern..

